# Evaluation of a Longitudinal Health Equity Curriculum: A Pilot Initiative for Advancing Inclusive Medical Education

**DOI:** 10.1177/23821205261477147

**Published:** 2026-08-10

**Authors:** Naseem Alammar, Yadira Rivera-Sanchez, Kelli Martinez, Whitney Cameron

**Affiliations:** 1 Division of Pediatric Hospital Medicine, University of Texas Southwestern Medical Center and Children’s Health, Dallas, Texas, US; 2 Division of Pediatric Pulmonology, University of Texas Southwestern Medical Center and Children’s Health, Dallas, Texas, US

**Keywords:** medical education, social determinants of health, health equity, health disparities, health inequities, curriculum development, pediatric hospital medicine

## Abstract

**Background:**

Despite the Accreditation Council for Graduate Medical Education (ACGME) requirements for education on health equity, many graduate medical education (GME) programs lack structured learning in this area. Health inequities persist in clinical practice, emphasizing the importance of incorporating training in health equity concepts.

**Objective:**

This study aimed to design and pilot a longitudinal health equity curriculum for pediatric fellows to improve their knowledge, beliefs, and comfort with equity-related concepts.

**Methods:**

A two-phase pilot study was conducted. Phase I was from October 2024–March 2025 within a two-year pediatric hospital medicine (PHM) fellowship. Of six total PHM fellows, five were eligible and participated. Curriculum development followed Kern’s six-step model. A needs assessment informed session topics. Four, sixty-minute interactive, in-person sessions were held. Phase II curriculum sessions were delivered to a separate replication cohort of pediatric fellows in November and December 2025. Anonymous pre-post-session surveys were administered to assess changes in knowledge, beliefs, and comfort. Demographic data was not collected during phase I to maintain anonymity but was collected from the larger phase II cohort. Results were compared using pre-post-session data, using descriptive statistics. Survey tools were internally developed and not externally validated.

**Results:**

During phase I, five fellows were eligible to participate, with 4-5 fellows per session (80-100% participation). All phase I participants (100%) completed both pre- and post-surveys. Self-reported knowledge, beliefs, and comfort improved across all sessions. In phase II, similar patterns of improvement were observed and participants shifted away from neutral or disagree responses, with marked improvements in knowledge, belief, and comfort domains.

**Conclusion:**

A longitudinal health equity curriculum was piloted within a PHM fellowship and then replicated in a larger cohort, with measurable improvements in understanding of health equity concepts. These findings support the integration of longitudinal health equity education into GME programs to better equip physicians to advance health equity in clinical practice.

## Introduction

Health inequities are the unfair and avoidable differences in health status and access to healthcare across different population groups, which are influenced by a variety of social, economic, and environmental determinants (drivers).^1^ Despite increasing awareness, health inequities remain prevalent in medicine and negatively impact outcomes like hospital cost, length of stay, pain control, and perioperative care.^2,3^ Addressing these disparities is essential, and the Accreditation Council for Graduate Medical Education (ACGME) now requires medical training to include education on how to address them.^4,5^ However, many medical educators find it challenging to implement effective curricula that prepare trainees to tackle the complex drivers of health inequity amidst changing institutional policies and state-level legislation.

While much literature exists on the impact of health inequities,^6,7^ fewer studies have evaluated educational interventions to address them, and the literature is inconsistent in how to measure the success of existing interventions. Longitudinal health equity curricula specifically designed for fellows remain scarce, and, to our knowledge, none have been developed or assessed for pediatric fellows—a group increasingly caring for diverse patient populations particularly at our institution.^8-11^ This study aimed to design and pilot a longitudinal health equity curriculum for pediatric fellows to improve their knowledge, beliefs, and comfort with equity-related concepts.

## Methods

The pilot was conducted in two phases. Phase I was conducted from October 2024 to March 2025, within a two-year PHM fellowship at a tertiary-care pediatric hospital in a major United States metropolitan area. Of six total PHM fellows, five were eligible and participated. The PHM fellow leading the curriculum development was excluded. Phase II was conducted in November 2025 and December 2025. Pediatric subspeciality fellows attending their existing academic half-day were eligible to participate in the voluntary sessions with sixty-seven total pediatric subspeciality fellows receiving the invite to participate. Demographic data was collected from the larger cohort of phase II participants. The same survey instruments were utilized during both phases with the addition of demographic questions incorporated into the phase II, curriculum session 1 pre-survey. The study was deemed exempt (research involving human subjects that qualifies for exemption due to minimal risk) by the University of Texas Southwestern institutional review board (IRB), and all materials underwent IRB review.

Phase I consisted of four, sixty-minute interactive, in-person sessions held throughout the academic year. The curriculum pilot was conducted within an existing educational block (an academic half-day schedule), requiring minimal additional time or resources beyond facilitator preparation. Curriculum development followed Kern’s six-step model,^12^ beginning with a literature review and a general needs assessment for PHM fellowship program leadership to identify the program’s overall needs. Then, a targeted needs assessment to identify the needs of our specific audience (pediatric fellows). Next, we created goals and objectives guided by the ACGME requirements on health equity. Measurable objectives were identified, which included beliefs, comfort, and knowledge as target outcomes. The session contents were created (N.A.) and reviewed by the curriculum development team. Our needs assessment informed session topics, which included health equity fundamentals (session one), cultural humility (session two), implicit bias (session three), and microaggressions (session four). Phase I sessions were led by the curriculum development team (N.A.) and used didactics, case discussions, multimedia, small-group discussions, and reflection questions.

During phase II, curriculum session one (health equity fundamentals) and session two (cultural humility) were delivered to pediatric subspeciality fellows over two hours during an existing academic half-day schedule. Demographic information was collected from phase II participants in the larger replication cohort. Results were analyzed using both Likert-scale descriptive statistics and proportional analysis of neutral/disagree responses to better characterize shifts in responses in a larger sample.

Anonymous pre- and post-session surveys were collected via RedCap to assess changes in knowledge, beliefs, and comfort. Surveys included self-assessment items rated on a 7-point Likert scale and multiple-choice knowledge questions tailored to session content. Results were compared using pre- and post-session data, using descriptive statistics; no statistical tests were performed due to the small sample size. Survey tools were internally developed by the curriculum development team and were not validated in this pilot study. (Supplemental material). The reporting of this study conforms to the Defined Criteria To Report INnovations in Education (DoCTRINE) (Supplemental material).^13^

## Results

Five PHM fellows participated in phase I and received curriculum sessions 1-4. The general needs assessment completed by the PHM program faculty leadership had a 100% response rate (n=2). Faculty members strongly agreed that receiving adequate training in health equity and health disparities was a priority for completion of training. The targeted (PHM fellow) needs assessment had an 80% response rate, which revealed that 75% had not previously experienced a longitudinal health equity curriculum and felt their prior training was inadequate. All participants in phase I completed pre- and post-session surveys for each of the four sessions, with 100% response rates and 80–100% session attendance. Demographic data was not collected during phase I to maintain anonymity. PHM Fellows responded positively, noting strong engagement and relevance to clinical practice. Self-reported knowledge, beliefs, and comfort improved across all sessions. The most notable gains were in knowledge, particularly in understanding health equity and addressing microaggressions as both recipients and bystanders (Table 1). Knowledge scores also improved in most sessions (Table 2). During phase II, thirty-two pediatric fellows participated in health equity fundamentals (curriculum session one), and twenty-six pediatric fellows participated in cultural humility (curriculum session two). Twenty-two participants identified as female, fifteen identified as white, and twenty-five as non-Hispanic (Table 3). Pre/post surveys were completed by 84% (27/32) of participants in curriculum session one and 69% (18/26) of participants in curriculum session two (Table 1). Pre- and post-session survey responses from the phase II cohort showed similar patterns of improvement on the Likert scale (Table 1). In curriculum session one, the proportion of participants reporting neutral or disagree responses regarding comfort with incorporating health equity techniques into daily practice decreased substantially, from 40.6% to 4.0%. Similar improvements were observed in comfort discussing health equity with patients (31.3% to 4.0%) and with colleagues (28.1% to 4.0%). In curriculum session two, uncertainty about how to practice cultural humility decreased (25.0% to 6.2%), and discomfort with discussing cultural humility with both colleagues and patients were eliminated following the session (Table 4).

**Table 1.** Participant Self-Reported Assessment of Knowledge, Beliefs, and Comfort Pre and Post Session With Pilot Cohort and Replication Cohort

​	Pilot cohort (phase 1)		Replication cohort (phase 2)	
	Pre-survey	Post-survey	Pre-survey	Post-survey
**Session 1: Introduction to Health Equity and Health Disparities**				
Number of Participants	5	5	32	27
**Knowledge** ^1^				
Examples of specific health inequities that exist in medicine	3	4	4	4
Impacts of health inequities on vulnerable populations	3	4	4	4
Specific health inequities that exist locally	2	4	3	4
Techniques to advance health equity in daily practice	2	4	3	4
**Beliefs** ^2^				
Non-medical factors can affect patient outcomes	7	7	7	7
Health inequities persist in medicine today and are a major problem	7	7	7	7
Physicians serve a primary role in advancing health equity	6	7	6	6
**Comfort** ^2^				
Discussing health equity with colleagues	6	6	5	6
Discussing health equity with patients	6	6	5	6
Incorporating techniques to advance health equity in daily practice	5	6	5	6
**Session 2: Cultural Humility**				
Number of Participants	4	4	26	18
**Knowledge** ^2^				
Importance of cultural humility and how it contributes to health equity	5	7	6	7
How to practice cultural humility with my patients	4.5	7	5	7
**Beliefs** ^2^				
Physicians should practice cultural humility	7	7	7	7
Lack of cultural humility in patient care can lead to poorer health outcomes	6.5	7	7	7
Cultural humility is crucial step to achieve health equity	6.5	7	7	7
**Comfort** ^2^				
Discussing cultural humility with colleagues	5.5	7	5	7
Discussing cultural humility with patients	5	6.5	5	7
**Session 3: Implicit Bias**				
Number of Participants	4	4	-	-
**Knowledge** ^2^				
Importance of implicit bias and how this contributes to health inequity	6	7	-	-
How to actively work to reduce bias and the impacts on patients	4	6	-	-
**Beliefs** ^2^	​	​	​	​
Everyone has bias	7	7	-	-
Implicit bias can negatively impact patient outcomes	6	6.5	-	-
Implicit bias is a major issue in healthcare and needs to be addressed	7	7	-	-
**Comfort** ^2^				
Discussing implicit bias with colleagues	6	7	-	-
Using techniques to reduce bias with my patients	5	6.5	-	-
**Session 4: Microaggressions**				
Number of Participants	5	5	​	​
**Knowledge** ^2^	​	​	​	​
Importance of microaggressions and how they contribute to health inequity	5	6	-	-
Frameworks to use to respond to microaggressions	4	6	-	-
Ability to notice when a microaggression occurs in the clinical environment	4	6	-	-
**Beliefs** ^2^				
Anyone can be a recipient, source, or bystander of a microaggression	7	7	-	-
Microaggressions occur frequently in the healthcare setting	6	6	-	-
Healthcare workers have an obligation to be aware of microaggressions	7	7	-	-
Physicians should know how to respond to microaggressions	7	6	-	-
**Comfort** ^2^				
Responding to a microaggression when a recipient	3	5	-	-
Responding to a microaggression when a bystander	3	5	-	-
Calling out a colleague who said a microaggression	5	5	-	-
Calling out a patient who said a microaggression	5	5	-	-

^1^Using a 5 point likert scale (1=not at all knowledgeable, 2= slightly knowledgeable, 3=moderately knowledgeable, 4= mostly knowledgeable, 5 fully knowledgeable). Most frequently reported responses are shown.

^2^Using a 7 point likert scale (1=strongly disagree, 2= moderately disagree, 3=disagree, 4= neutral, 5= agree, 6= moderately agree, 7=strongly agree). Most frequently reported responses are shown.

**Table 2.** Comparison of Scores on Knowledge-Based Questions Prior to and After Each Respective Session

​	Pilot cohort (phase 1)		Replication cohort (phase 2)	
	Pre-session knowledge score	Post-session knowledge score	Pre-session knowledge score	Post-session knowledge score
Session 1	50%	75%	50%	73%
Session 2	100%	100%	90%	91.6%
Session 3	94%	100%	-	-
Session 4	80%	87%	-	-

**Table 3.** Demographic Information From Phase II Pediatric Fellow Participants

Gender	N= 32 (%)
Female	22 (69)
Male	8 (25)
Non-Binary	**0 (0)**
Other	**0 (0)**
Prefer not to answer	**0 (0)**
**Race**	
American Indian	0 (0)
Asian or Asian American	7 (22)
Black, African, or African American	3 (9)
Latino or of Spanish Origin	5 (15)
Middle Eastern or North African	2 (6)
Native Hawaiian or other Pacific Islander	0 (0)
White	15 (46)
Prefer Not to answer	2 (6)
**Ethnicity**	
Hispanic	5 (15)
Non-Hispanic	25 (78)
Prefer not to answer	2 (6

**Table 4.** Changes in Proportion of Participants Reporting Neutral or Disagreement Pre- and Post-session in Larger Replication Cohort

Session	Topic	Pre-survey (Disagree/Neutral %)	Post-survey (Disagree/Neutral %)	Total improvement
Session 1	Knowledgeable in examples of specific health inequities	6.5%	0%	+6.5%
Session 1	Knowledgeable in impacts of health inequities on vulnerable populations	6.3%	0%	+6.3%
Session 1	Knowledgeable in health inequities that exist locally	31.3%	0%	+31.3%
Session 1	Knowledgeable in techniques to advance health equity in daily practice	31.3%	0%	+31.3%
Session 1	Belief that non-medical factors can affect patient outcomes	9.4%	0%	+9.4%
Session 1	Belief that health inequities persist in medicine today	12.5%	4.0%	+8.5%
Session 1	Belief that physicians serve a primary role in advance health equity	12.5%	4.0%	+8.5%
Session 1	Comfort in discussing health equity with patients	31.3%	4.0%	+27.3%
Session 1	Comfort in discussing health equity with colleagues	28.1%	4.0%	+24.1%
Session 1	Comfort in incorporating techniques to advance health equity in daily practice	40.6%	4.0%	+36.6%
Session 2	Knowledgeable of importance cultural humility	25%	12.5%	+12.5%
Session 2	Knowledgeable on how to practice cultural humility	25%	6.25%	+18.75%
Session 2	Belief physicians should practice cultural humility	4.2%	0%	+4.2%
Session 2	Belief lack of cultural humility in patient care can lead to poorer health outcomes	4.2%	0%	+4.2%
Session 2	Belief cultural humility is crucial step to achieve health equity	8.3%	6.25%	+2.05%
Session 2	Comfort practicing cultural humility with patients	12.5%	0.0%	+12.5%
Session 2	Comfort discussing cultural humility with colleagues	16.7%	0.0%	+16.7%

## Discussion

This pilot study found that a longitudinal health equity curriculum for pediatric fellows was associated with increased knowledge, beliefs, and comfort regarding key equity topics. Most fellows reported inadequate prior exposure to health equity education, despite training in a county where 21% of children live below the poverty line, of which 27 and 24% of impoverished children identify as Black or Hispanic, respectively.^14^ Knowledge improvements were both perceived by participants and through objective assessments. Delivery of curriculum sessions one and two to a separate, larger cohort demonstrated similar patterns of improvement, with a reduction in the proportion of participants reporting neutral or disagree responses across multiple domains. These findings suggest that the observed effects of the curriculum are reproducible across different learner groups and settings within the same training environment. The consistency of improvements across cohorts further supports the potential for broader implementation of this curriculum within graduate medical education programs. Longitudinal delivery may have helped build deeper conceptual understanding than one-time sessions.^15^ While this was a small study without formal statistical analysis, the descriptive results are consistent with prior research showing that structured curricula can positively impact trainee engagement with health equity concepts.^16,17^ These findings add to a small but growing body of literature demonstrating that equity-focused education can be successfully integrated into GME when designed thoughtfully and aligned with existing educational structures using minimal additional resources.

### Limitations and Future Directions

This study had several limitations. The study involved a small, single-institution sample size, limiting generalizability. No statistical analysis was performed because of the small sample size of the pilot group. Survey instruments were not validated as part of this pilot, and there was no long-term follow-up to assess sustained impact or behavioral change. Improvements in beliefs and comfort were self-reported and not tied to observed clinical behavior or decision-making. Future directions include a formal implementation study, expanding this curriculum to more training programs and subspecialties, assessing long-term effects on clinical practice, and validation of survey instruments. Larger studies could also support statistical analysis to better quantify the curriculum’s impact.

## Conclusion

A longitudinal health equity curriculum was piloted within a pediatric hospital medicine fellowship and subsequently reproduced in a larger, cohort. Both groups demonstrated improved knowledge, beliefs, and comfort with key health equity concepts. The consistency of findings across cohorts supports the reproducibility and scalability of this curriculum. These findings support the value of embedding health equity education into training programs to better prepare physicians to address healthcare disparities.

## Supplemental Material

Supplemental material - Evaluation of a Longitudinal Health Equity Curriculum: A Pilot Initiative for Advancing Inclusive Medical Education Supplemental material for Evaluation of a Longitudinal Health Equity Curriculum: A Pilot Initiative for Advancing Inclusive Medical Education by Naseem Alammar, Yadira Rivera-Sanchez, Kelli Martinez, Whitney Cameron in Journal of Medical Education and Curricular Development.

Supplemental material - Evaluation of a Longitudinal Health Equity Curriculum: A Pilot Initiative for Advancing Inclusive Medical Education Supplemental material for Evaluation of a Longitudinal Health Equity Curriculum: A Pilot Initiative for Advancing Inclusive Medical Education by Naseem Alammar, Yadira Rivera-Sanchez, Kelli Martinez, Whitney Cameron in Journal of Medical Education and Curricular Development.
