# Supplementary material for: Evaluation of a Longitudinal Health Equity Curriculum: A Pilot Initiative for Advancing Inclusive Medical Education
Source: J Med Educ Curric Dev. 2026 Aug 10;13:23821205261477147. doi: 10.1177/23821205261477147 (PMC13458116; doi:10.1177/23821205261477147)
Supplement: Supplementary file 1 [file sj-pdf-1-mde-10.1177_23821205261477147.pdf]

Landing Page

Please complete the survey below.

Thank you!

Thank you for participating in this health equity longitudinal curriculum study! We appreciate your time and feedback!
Click "submit" below to move onto the next page to fill out the survey.

Session 1 Pre Survey

Please complete the survey below.

Thank you!

What is your gender identity?

- ◯ Female
- ◯ Male
- ◯ Non-binary
- ◯ Other
- ◯ Prefer not to answer

What is your race? Please select all that apply

- ☐ American Indian or Alaska Native
- ☐ Asian or Asian American
- ☐ Black, African or African American
- ☐ Latino, or of Spanish origin
- ☐ Middle Eastern or North African
- ☐ Native Hawaiian or other Pacific Islander
- ☐ White
- ☐ Prefer not to answer

What is your ethnicity?

- ◯ Hispanic
- ◯ Non-Hispanic
- ◯ Prefer not to answer

How many years have you been in practice?

- ◯ Trainee
- ◯ 1-5
- ◯ 6-10
- ◯ 10-15
- ◯ 15+

Which of the following best defines health disparities?

- ◯ Differences in health outcomes that are primarily caused by individual lifestyle choices, such as diet and exercise
- ◯ The unequal distribution of healthcare services based on income and insurance status
- ◯ A particular type of health difference that is closely linked with economic, social, or environmental disadvantage
- ◯ Health differences that stem from unjust, systemic policies and practices which limit opportunities for good health

---

Which of the following best defines health inequities?

- ◯ Differences in health outcomes that are primarily caused by individual lifestyle choices, such as diet and exercise
- ◯ The unequal distribution of healthcare services based on income and insurance status
- ◯ A particular type of health difference that is closely linked with economic, social, or environmental disadvantage
- ◯ Health differences that stem from unjust, systemic policies and practices which limit opportunities for good health

---

Which of the following best defines health equity?

- ◯ State in which everyone has an equal opportunity to attain their highest level of health
- ◯ State in which everyone has a fair opportunity to attain their highest level of health
- ◯ The idea that all individuals should have access to healthcare regardless of their geographical location or socio-economic status
- ◯ The belief that everyone should have equal access to healthcare services, regardless of their ability to pay.

Which of the following factors contribute to health inequities:

◯ Socioeconomic Status
◯ Language Barriers
◯ Access to Healthcare
◯ Environmental Conditions
◯ Genetics
◯ All of the above
◯ Socioeconomic Status, Language Barriers, and Access to Healthcare
◯ Socioeconomic Status, Language Barriers, Access to Healthcare, and Environmental Conditions

Please rate your level of knowledge on a scale of 1-5 with 1 being no knowledge at all and 5 being fully knowledgeable

	1	2	3	4	5
Examples of specific health inequities that exist in medicine	◯	◯	◯	◯	◯
The impacts of health inequities on vulnerable populations	◯	◯	◯	◯	◯
Examples of specific health inequities that exist in Dallas	◯	◯	◯	◯	◯
Techniques to advance health equity in daily practice	◯	◯	◯	◯	◯

Please rate your beliefs:

	Strongly Disagree	Moderately Disagree	Slightly Disagree	Neutral	Slightly Agree	Moderately Agree	Strongly Agree
I believe non-medical factors can affect patient outcomes	◯	◯	◯	◯	◯	◯	◯
I believe health inequities persist in medicine today and are a major problem	◯	◯	◯	◯	◯	◯	◯
Although I believe health inequities persist in medicine today, I believe it is less of a problem today than previously	◯	◯	◯	◯	◯	◯	◯
I believe physicians serve a primary role in advancing health equity	◯	◯	◯	◯	◯	◯	◯

Please rate your comfort

	Strongly Disagree	Moderately Disagree	Slightly Disagree	Neutral	Slightly Agree	Moderately Agree	Strongly Agree
I feel comfortable discussing health equity concepts with my colleagues	◯	◯	◯	◯	◯	◯	◯

I feel comfortable discussing factors that may contribute to health inequities with my patients	◯	◯	◯	◯	◯	◯	◯
I feel comfortable incorporating techniques to advance health equity in my daily practice	◯	◯	◯	◯	◯	◯	◯

Session 1 Post Survey

Please complete the survey below.

Thank you!

Which of the following best defines health disparities?

- ◯ Differences in health outcomes that are primarily caused by individual lifestyle choices, such as diet and exercise
- ◯ The unequal distribution of healthcare services based on income and insurance status
- ◯ A particular type of health difference that is closely linked with economic, social, or environmental disadvantage
- ◯ Health differences that stem from unjust, systemic policies and practices which limit opportunities for good health

---

Which of the following best defines health inequities?

- ◯ Differences in health outcomes that are primarily caused by individual lifestyle choices, such as diet and exercise
- ◯ The unequal distribution of healthcare services based on income and insurance status
- ◯ A particular type of health difference that is closely linked with economic, social, or environmental disadvantage
- ◯ Health differences that stem from unjust, systemic policies and practices which limit opportunities for good health

---

Which of the following best defines health equity?

- ◯ State in which everyone has an equal opportunity to attain their highest level of health
- ◯ State in which everyone has a fair opportunity to attain their highest level of health
- ◯ The idea that all individuals should have access to healthcare regardless of their geographical location or socio-economic status
- ◯ The belief that everyone should have equal access to healthcare services, regardless of their ability to pay.

Which of the following factors contribute to health inequities:

- ◯ Socioeconomic Status
- ◯ Language Barriers
- ◯ Access to Healthcare
- ◯ Environmental Conditions
- ◯ Genetics
- ◯ All of the above
- ◯ Socioeconomic Status, Language Barriers, and Access to Healthcare
- ◯ Socioeconomic Status, Language Barriers, Access to Healthcare, and Environmental Conditions

Please rate your level of knowledge on a scale of 1-5 with 1 being no knowledge at all and 5 being fully knowledgeable

	1	2	3	4	5
Examples of specific health inequities that exist in medicine	◯	◯	◯	◯	◯
Examples of specific health inequities that exist in Dallas	◯	◯	◯	◯	◯
The impact of health inequities on vulnerable populations	◯	◯	◯	◯	◯

Techniques to advance health equity in daily practice	○	○	○	○	○

Please rate your beliefs:

	Strongly Disagree	Moderately Disagree	Slightly Disagree	Neutral	Slightly Agree	Moderately Agree	Strongly Agree
I believe non-medical factors can affect patient outcomes	○	○	○	○	○	○	○
I believe health inequities persist in medicine today and are a major problem	○	○	○	○	○	○	○
Although I believe health inequities persist in medicine today, I believe it is less of a problem today than previously	○	○	○	○	○	○	○
I believe physicians serve a primary role in advancing health equity	○	○	○	○	○	○	○

Please rate your comfort

	Strongly Disagree	Moderately Disagree	Slightly Disagree	Neutral	Slightly Agree	Moderately Agree	Strongly Agree
I feel comfortable discussing health equity concepts with my colleagues	○	○	○	○	○	○	○
I feel comfortable discussing factors that may contribute to health inequities with my patients	○	○	○	○	○	○	○
I feel comfortable incorporating techniques to advance health equity in my daily practice	○	○	○	○	○	○	○

Session 2 Pre Survey

Please complete the survey below.

Thank you!

What is your gender identity?	◯ Female<br>◯ Male<br>◯ Non-binary<br>◯ Other<br>◯ Prefer not to answer
What is your race? Please select all that apply	☐ American Indian or Alaska Native<br>☐ Asian or Asian American<br>☐ Black, African or African American<br>☐ Latino, or of Spanish origin<br>☐ Middle Eastern or North African<br>☐ Native Hawaiian or other Pacific Islander<br>☐ White<br>☐ Prefer not to answer
What is your ethnicity?	◯ Hispanic<br>◯ Non-Hispanic<br>◯ Prefer not to answer
How many years have you been in practice?	◯ Trainee<br>◯ 1-5<br>◯ 6-10<br>◯ 10-15<br>◯ 15+
Which of the following best defines cultural competency?	◯ The ability of an individual to understand and respect values, attitudes, and beliefs that differ across cultures, and to consider and respond appropriately to these differences<br>◯ Admitting that one does not know and is willing to learn from patients about their experiences, while being aware of one's own embeddedness in culture<br>◯ The ability to be aware of and accepting of cultural differences, and to work well with people from other cultures<br>◯ The ability to treat all patients exactly the same, without considering cultural differences.
Which of the following best defines cultural humility?	◯ The ability of an individual to understand and respect values, attitudes, and beliefs that differ across cultures, and to consider and respond appropriately to these differences<br>◯ Admitting that one does not know and is willing to learn from patients about their experiences, while being aware of one's own embeddedness in culture<br>◯ The ability to be aware of and accepting of cultural differences, and to work well with people from other cultures<br>◯ The ability to treat all patients exactly the same, without considering cultural differences.

Which of the following best explains the difference between cultural competency and cultural humility?

- ◯ Cultural humility is a lifelong process where you admit you do not know everything about every culture, while cultural competency is an end product where you are the expert that knows everything
- ◯ Cultural competency is a lifelong process where you admit you do not know everything about every culture, while cultural humility is an end product where you are the expert that knows everything

I am knowledgeable on the importance of cultural humility and how lack of this can contribute to health inequity

- ◯ Strongly Agree
- ◯ Moderately Agree
- ◯ Agree
- ◯ Neutral
- ◯ Disagree
- ◯ Moderately Disagree
- ◯ Strongly Disagree

I am knowledgeable on how I can practice cultural humility with my patients

- ◯ Strongly Agree
- ◯ Moderately Agree
- ◯ Agree
- ◯ Neutral
- ◯ Disagree
- ◯ Moderately Disagree
- ◯ Strongly Disagree

Please rate your beliefs

	Strongly Disagree	Mod. Disagree	Disagree	Neutral	Agree	Mod. Agree	Strongly Agree
I believe as physicians, we should be culturally competent	◯	◯	◯	◯	◯	◯	◯
I believe as physicians, we do not necessarily need to be fully culturally competent	◯	◯	◯	◯	◯	◯	◯
I believe as physicians, we should practice cultural humility	◯	◯	◯	◯	◯	◯	◯
I believe lack of cultural humility in patient care can lead to poorer health outcomes	◯	◯	◯	◯	◯	◯	◯
I believe cultural humility is a crucial step to achieve health equity	◯	◯	◯	◯	◯	◯	◯

Please rate your comfort:

	Strongly Disagree	Mod. Disagree	Disagree	Neutral	Agree	Mod. Agree	Strongly Agree
I feel comfortable discussing cultural humility with my colleagues	◯	◯	◯	◯	◯	◯	◯

I feel comfortable practicing cultural humility with my patients	○	○	○	○	○	○	○

Session 2 Post Survey

Please complete the survey below.

Thank you!

---

51) Which of the following best defines cultural competency?

- ◯ The ability of an individual to understand and respect values, attitudes, and beliefs that differ across cultures, and to consider and respond appropriately to these differences
- ◯ Admitting that one does not know and is willing to learn from patients about their experiences, while being aware of one's own embeddedness in culture
- ◯ The ability to be aware of and accepting of cultural differences, and to work well with people from other cultures
- ◯ The ability to treat all patients exactly the same, without considering cultural differences.

---

52) Which of the following best defines cultural humility?

- ◯ The ability of an individual to understand and respect values, attitudes, and beliefs that differ across cultures, and to consider and respond appropriately to these differences
- ◯ Admitting that one does not know and is willing to learn from patients about their experiences, while being aware of one's own embeddedness in culture
- ◯ The ability to be aware of and accepting of cultural differences, and to work well with people from other cultures
- ◯ The ability to treat all patients exactly the same, without considering cultural differences.

---

53) Which of the following best explains the difference between cultural competency and cultural humility?

- ◯ Cultural humility is a lifelong process where you admit you do not know everything about every culture, while cultural competency is an end product where you are the expert that knows everything
- ◯ Cultural competency is a lifelong process where you admit you do not know everything about every culture, while cultural humility is an end product where you are the expert that knows everything

---

54) I am knowledgeable on the importance of cultural humility and how lack of this can contribute to health inequity

- ◯ Strongly Agree
- ◯ Moderately Agree
- ◯ Agree
- ◯ Neutral
- ◯ Disagree
- ◯ Moderately Disagree
- ◯ Strongly Disagree

---

55) I am knowledgeable on how I can practice cultural humility with my patients

- ◯ Strongly Agree
- ◯ Moderately Agree
- ◯ Agree
- ◯ Neutral
- ◯ Disagree
- ◯ Moderately Disagree
- ◯ Strongly Disagree

Please rate your beliefs

	Strongly Disagree	Mod. Disagree	Disagree	Neutral	Agree	Mod. Agree	Strongly Agree
56) I believe as physicians, we should be culturally competent	○	○	○	○	○	○	○
57) I believe as physicians, we do not necessarily need to be fully culturally competent	○	○	○	○	○	○	○
58) I believe as physicians, we should practice cultural humility	○	○	○	○	○	○	○
59) I believe lack of cultural humility in patient care can lead to poorer health outcomes	○	○	○	○	○	○	○
60) I believe cultural humility is a crucial step to achieve health equity	○	○	○	○	○	○	○

Please rate your comfort:

	Strongly Disagree	Mod. Disagree	Disagree	Neutral	Agree	Mod. Agree	Strongly Agree
61) I feel comfortable discussing cultural humility with my colleagues	○	○	○	○	○	○	○
62) I feel comfortable practicing cultural humility with my patients	○	○	○	○	○	○	○

Session 3 Pre Survey

Please complete the survey below.

Thank you!

---

63) Which of the following best defines implicit bias?

- ◯ A conscious preference for one group over another
- ◯ An automatic and unconscious attitude towards a group or individual
- ◯ A bias that is always deliberate and intentional
- ◯ A belief that all people are equally capable and deserving

---

64) What is the difference between implicit and explicit bias?

- ◯ Implicit bias is expressed indirectly and operates subconsciously while explicit bias is expressed directly and operates consciously
- ◯ Implicit bias is expressed directly and operates consciously while explicit bias is expressed indirectly and operates subconsciously
- ◯ Implicit bias only affects individuals from minority groups, while explicit bias affects everyone
- ◯ Implicit bias involves overt actions while explicit bias only influences thoughts

---

65) Which of the following is a true statement?

- ◯ Not everyone has biases, but those that do must work hard to ensure it does not affect patient care
- ◯ Only individuals from certain backgrounds have biases
- ◯ Biases only affect how people are treated from different cultures
- ◯ Everyone has biases; however, they can be unlearned with effort and awareness

---

66) Which of the following is NOT true about how you can work to unlearn implicit bias

- ◯ Unlearning unconscious bias requires ongoing self-awareness and effort over time
- ◯ Acknowledging and identifying biases is enough to eliminate them
- ◯ Actively countering stereotypes and asking questions
- ◯ Regularly reflecting on personal biases and engaging in active reflection

---

67) I am knowledgeable on the importance of implicit bias and how this can contribute to health inequity

- ◯ Strongly Agree
- ◯ Moderately Agree
- ◯ Agree
- ◯ Neutral
- ◯ Disagree
- ◯ Moderately Disagree
- ◯ Strongly Disagree

---

68) I am knowledgeable on how I can actively work to reduce implicit bias to lessen the impacts on my patients

- ◯ Strongly Agree
- ◯ Moderately Agree
- ◯ Agree
- ◯ Neutral
- ◯ Disagree
- ◯ Moderately Disagree
- ◯ Strongly Disagree

Please rate your beliefs:

		Strongly Disagree	Moderately Disagree	Disagree	Neutral	Agree	Moderately Agree	Strongly Agree
69)	I believe everyone has biases	○	○	○	○	○	○	○
70)	Having biases makes you a bad person	○	○	○	○	○	○	○
71)	I believe implicit biases can negatively impact patient outcomes	○	○	○	○	○	○	○
72)	I believe implicit bias is a major issue in healthcare and needs to be addressed	○	○	○	○	○	○	○
73)	Although implicit bias is a major issue in healthcare, I believe explicit bias is not an issue	○	○	○	○	○	○	○

Please rate your comfort:

		Strongly Disagree	Moderately Disagree	Disagree	Neutral	Agree	Moderately Agree	Strongly Agree
74)	I feel comfortable discussing implicit bias with my colleagues	○	○	○	○	○	○	○
75)	I feel comfortable using techniques to reduce implicit bias with my patients	○	○	○	○	○	○	○

Session 3 Post Survey

Please complete the survey below.

Thank you!

---

76) Which of the following best defines implicit bias?

- ◯ A conscious preference for one group over another
- ◯ An automatic and unconscious attitude towards a group or individual
- ◯ A bias that is always deliberate and intentional
- ◯ A belief that all people are equally capable and deserving

---

77) What is the difference between implicit and explicit bias?

- ◯ Implicit bias is expressed indirectly and operates subconsciously while explicit bias is expressed directly and operates consciously
- ◯ Implicit bias is expressed directly and operates consciously while explicit bias is expressed indirectly and operates subconsciously
- ◯ Implicit bias only affects individuals from minority groups, while explicit bias affects everyone
- ◯ Implicit bias involves overt actions while explicit bias only influences thoughts

---

78) Which of the following is a true statement?

- ◯ Not everyone has biases, but those that do must work hard to ensure it does not affect patient care
- ◯ Only individuals from certain backgrounds have biases
- ◯ Biases only affect how people are treated from different cultures
- ◯ Everyone has biases; however, they can be unlearned with effort and awareness

---

79) Which of the following is NOT true about how you can work to unlearn implicit bias

- ◯ Unlearning unconscious bias requires ongoing self-awareness and effort over time
- ◯ Acknowledging and identifying biases is enough to eliminate them
- ◯ Actively countering stereotypes and asking questions
- ◯ Regularly reflecting on personal biases and engaging in active reflection

---

80) I am knowledgeable on the importance of implicit bias and how this can contribute to health inequity

- ◯ Strongly Agree
- ◯ Moderately Agree
- ◯ Agree
- ◯ Neutral
- ◯ Disagree
- ◯ Moderately Disagree
- ◯ Strongly Disagree

---

81) I am knowledgeable on how I can actively work to reduce implicit bias to lessen the impacts on my patients

- ◯ Strongly Agree
- ◯ Moderately Agree
- ◯ Agree
- ◯ Neutral
- ◯ Disagree
- ◯ Moderately Disagree
- ◯ Strongly Disagree

Please rate your beliefs:

		Strongly Disagree	Moderately Disagree	Disagree	Neutral	Agree	Moderately Agree	Strongly Agree
82)	I believe everyone has biases	○	○	○	○	○	○	○
83)	Having biases makes you a bad person	○	○	○	○	○	○	○
84)	I believe implicit biases can negatively impact patient outcomes	○	○	○	○	○	○	○
85)	I believe implicit bias is a major issue in healthcare and needs to be addressed	○	○	○	○	○	○	○
86)	Although implicit bias is a major issue in healthcare, I believe explicit bias is not an issue	○	○	○	○	○	○	○

Please rate your comfort:

		Strongly Disagree	Moderately Disagree	Disagree	Neutral	Agree	Moderately Agree	Strongly Agree
87)	I feel comfortable discussing implicit bias with my colleagues	○	○	○	○	○	○	○
88)	I feel comfortable using techniques to reduce implicit bias with my patients	○	○	○	○	○	○	○

Session 4 Pre Survey

Please complete the survey below.

Thank you!

Which of the following best defines microaggressions?

- ◯ Large-scale acts of discrimination that are overt and intentional.
- ◯ often unintentional, verbal or behavioral slights that perpetuate stereotypes and hurt marginalized groups.
- ◯ Conscious efforts to challenge and oppose prejudiced behavior.
- ◯ Aggressive actions taken by individuals in response to feeling marginalized or discriminated against.

What of the following is a true statement?

- ◯ Microassaults are always unintentional
- ◯ Microassaults are deliberate and intentional attacks
- ◯ Microinsults involve direct verbal slurs
- ◯ Microinsults are subtle, often unintentional slights
- ◯ Microassaults, microinsults, and microinvalidations are all primarily concerned with promoting positive stereotypes.
- ◯ Microinvalidations dismiss or invalidate the experiences of marginalized individuals.
- ◯ A and C
- ◯ B, D, and F
- ◯ All of the above

Which of the following is NOT a microaggression?

- ◯ Telling a Latina colleague, "You're lucky to have a job; there are so many people of your background who don't even try to get hired."
- ◯ Asking a person with a disability, "What happened to you?" in a way that implies they are defined solely by their condition.
- ◯ Complimenting a Black colleague's "perfect English" despite knowing they are a native speaker of the language.
- ◯ Saying to a Black person, "I don't see color, we're all the same."

Can microaggressions occur without the perpetrator intending to offend the individual?

- ◯ Yes, they can occur unintentionally
- ◯ No, they only happen intentionally
- ◯ I'm not sure

I am knowledgeable on the importance of microaggressions in healthcare and how this can contribute to health inequity

- ◯ Strongly Agree
- ◯ Moderately Agree
- ◯ Agree
- ◯ Neutral
- ◯ Disagree
- ◯ Moderately Disagree
- ◯ Strongly Disagree

I am knowledgeable on frameworks I can use to respond to microaggressions in the moment

◯ Strongly Agree
◯ Moderately Agree
◯ Agree
◯ Neutral
◯ Disagree
◯ Moderately Disagree
◯ Strongly Disagree

I feel confident in my ability to notice when a microaggression is occurring in the clinical environment

◯ Strongly Agree
◯ Moderately Agree
◯ Agree
◯ Neutral
◯ Disagree
◯ Moderately Disagree
◯ Strongly Disagree

Please rate your beliefs:

	Strongly Disagree	Moderately Disagree	Disagree	Neutral	Agree	Moderately Agree	Strongly Agree
I believe anyone can be a recipient, source, or bystander of a microaggression	◯	◯	◯	◯	◯	◯	◯
I believe microaggressions occur frequently in the healthcare setting	◯	◯	◯	◯	◯	◯	◯
I believe healthcare workers have an obligation to be aware of microaggressions in the clinical environment	◯	◯	◯	◯	◯	◯	◯
I believe physicians should know how to respond to microaggressions in the moment	◯	◯	◯	◯	◯	◯	◯

Please rate your comfort:

	Strongly Disagree	Moderately Disagree	Disagree	Agree	Moderately Agree	Strongly Agree
I feel comfortable responding to a microaggression in the clinical environment when I'm a recipient	◯	◯	◯	◯	◯	◯
I feel comfortable responding to a microaggression in the clinical environment when I'm a bystander	◯	◯	◯	◯	◯	◯
I feel comfortable confronting or calling out a colleague who said a microaggression even if it is unintentional	◯	◯	◯	◯	◯	◯

I feel comfortable confronting or calling out a patient who said a microaggression even if it is unintentional	○	○	○	○	○	○

Session 4 Post Survey

Please complete the survey below.

Thank you!

Which of the following best defines microaggressions?

- ◯ Large-scale acts of discrimination that are overt and intentional.
- ◯ often unintentional, verbal or behavioral slights that perpetuate stereotypes and hurt marginalized groups.
- ◯ Conscious efforts to challenge and oppose prejudiced behavior.
- ◯ Aggressive actions taken by individuals in response to feeling marginalized or discriminated against.

What of the following is a true statement?

- ◯ Microassaults are always unintentional
- ◯ Microassaults are deliberate and intentional attacks
- ◯ Microinsults involve direct verbal slurs
- ◯ Microinsults are subtle, often unintentional slights
- ◯ Microassaults, microinsults, and microinvalidations are all primarily concerned with promoting positive stereotypes.
- ◯ Microinvalidations dismiss or invalidate the experiences of marginalized individuals.
- ◯ A and C
- ◯ B, D, and F
- ◯ All of the above

Which of the following is NOT an example of a microaggression

- ◯ Telling a Latina colleague, "You're lucky to have a job; there are so many people of your background who don't even try to get hired."
- ◯ Asking a person with a disability, "What happened to you?" in a way that implies they are defined solely by their condition.
- ◯ Complimenting a Black colleague's "perfect English" despite knowing they are a native speaker of the language.
- ◯ Saying to a Black person, "I don't see color, we're all the same."

Can microaggressions occur without the perpetrator intending to offend the individual?

- ◯ Yes, they can occur unintentionally
- ◯ No, they only happen intentionally
- ◯ I'm not sure

I am knowledgeable on the importance of microaggressions in healthcare and how this can contribute to health inequity

- ◯ Strongly Agree
- ◯ Moderately Agree
- ◯ Agree
- ◯ Neutral
- ◯ Disagree
- ◯ Moderately Disagree
- ◯ Strongly Disagree

I am knowledgeable on frameworks I can use to respond to microaggressions in the moment

◯ Strongly Agree
◯ Moderately Agree
◯ Agree
◯ Neutral
◯ Disagree
◯ Moderately Disagree
◯ Strongly Disagree

I feel confident in my ability to notice when a microaggression is occurring in the clinical environment

◯ Strongly Agree
◯ Moderately Agree
◯ Agree
◯ Neutral
◯ Disagree
◯ Moderately Disagree
◯ Strongly Disagree

Please rate your beliefs:

	Strongly Disagree	Moderately Disagree	Disagree	Neutral	Agree	Moderately Agree	Strongly Agree
I believe anyone can be a recipient, source, or bystander of a microaggression	◯	◯	◯	◯	◯	◯	◯
I believe microaggressions occur frequently in the healthcare setting	◯	◯	◯	◯	◯	◯	◯
I believe healthcare workers have an obligation to be aware of microaggressions in the clinical environment	◯	◯	◯	◯	◯	◯	◯
I believe physicians should know how to respond to microaggressions in the moment	◯	◯	◯	◯	◯	◯	◯

Please rate your comfort:

	Strongly Disagree	Moderately Disagree	Disagree	Agree	Moderately Agree	Strongly Agree
I feel comfortable responding to a microaggression in the clinical environment when I'm a recipient	◯	◯	◯	◯	◯	◯
I feel comfortable responding to a microaggression in the clinical environment when I'm a bystander	◯	◯	◯	◯	◯	◯
I feel comfortable confronting or calling out a colleague who said a microaggression even if it is unintentional	◯	◯	◯	◯	◯	◯

I feel comfortable confronting or calling out a patient who said a microaggression even if it is unintentional	◯	◯	◯	◯	◯	◯

Health Equity Curriculum Needs Assessment

Please complete the survey below.

Thank you!

What is your gender identity?

◯ Female
◯ Male
◯ Non-binary
◯ Other
◯ Prefer not to answer

What is your race? Please select all that apply

☐ American Indian or Alaska Native
☐ Asian or Asian American
☐ Black, African or African American
☐ Latino, or of Spanish origin
☐ Middle Eastern or North African
☐ Native Hawaiian or other Pacific Islander
☐ White
☐ Prefer not to answer

What is your ethnicity?

◯ Hispanic
◯ Non-Hispanic
◯ Prefer not to answer

What is your current role?

◯ Fellow
◯ Faculty
◯ Fellowship Program Leader

How many years have you been in practice?

◯ Trainee
◯ 1-5
◯ 6-10
◯ 10-15
◯ 15+

I have participated in a longitudinal health equity curriculum previously

◯ Yes
◯ No

If so, where? What did the curriculum entail? Please be specific!

____

I have participated in a short term health equity session previously (including single workshops, didactics, grand rounds, etc)

◯ Yes
◯ No

If so, where? What did this session specifically entail? Please be specific!

____

I believe I have received adequate education on health equity and health disparities.

◯ Strongly Agree
◯ Somewhat Agree
◯ Neither Agree Nor Disagree
◯ Somewhat Disagree
◯ Strongly Disagree

I believe I have received adequate education on cultural humility and cultural competency.

◯ Strongly Agree
◯ Somewhat Agree
◯ Neither Agree Nor Disagree
◯ Somewhat Disagree
◯ Strongly Disagree

I believe I have received adequate education on implicit and unconscious bias.	◯ Strongly Agree<br>◯ Somewhat Agree<br>◯ Neither Agree Nor Disagree<br>◯ Somewhat Disagree<br>◯ Strongly Disagree
I know how to reduce unconscious bias and feel comfortable addressing this in the workplace.	◯ Strongly Agree<br>◯ Somewhat Agree<br>◯ Neither Agree Nor Disagree<br>◯ Somewhat Disagree<br>◯ Strongly Disagree
I believe I have received adequate education on social determinants of health.	◯ Strongly Agree<br>◯ Somewhat Agree<br>◯ Neither Agree Nor Disagree<br>◯ Somewhat Disagree<br>◯ Strongly Disagree
I feel comfortable discussing social determinants of health with my patients and feel comfortable addressing their identified needs	◯ Strongly Agree<br>◯ Somewhat Agree<br>◯ Neither Agree Nor Disagree<br>◯ Somewhat Disagree<br>◯ Strongly Disagree
I would like to learn more about the following health equity topics:	______________________________

According to the Pediatric Hospital Medicine Fellowship ACGME Requirements, "Programs must understand the social determinants of health of the populations they serve and incorporate them in the design and implementation of the program curriculum, with the ultimate goal of addressing these needs and health disparities." With this in mind, please answer the following questions:

I believe the current curriculum includes adequate health equity and health disparity training for pediatric hospital medicine fellows.	◯ Strongly Agree<br>◯ Somewhat Agree<br>◯ Neither Agree Nor Disagree<br>◯ Somewhat Disagree<br>◯ Strongly Disagree
I believe it is important for our fellows to receive adequate training in health equity and health disparities.	◯ Strongly Agree<br>◯ Somewhat Agree<br>◯ Neither Agree Nor Disagree<br>◯ Somewhat Disagree<br>◯ Strongly Disagree
I would like the following health equity topics to be incorporated into the curriculum:	______________________________
