# Supplementary material for: Evaluation of a Longitudinal Health Equity Curriculum: A Pilot Initiative for Advancing Inclusive Medical Education
Source: J Med Educ Curric Dev. 2026 Aug 10;13:23821205261477147. doi: 10.1177/23821205261477147 (PMC13458116; doi:10.1177/23821205261477147)
Supplement: Supplementary file 2 [file sj-pdf-2-mde-10.1177_23821205261477147.pdf]

Defined Criteria To Report INnovations in Education (DoCTRINE)

Citation: Blanco M, Prunuske J, DiCorcia M, Learman LA, Mutcheson B, Huang GC. The DoCTRINE Guidelines: Defined Criteria To Report INnovations in Education. Academic Medicine. 2022 May 1;97(5):689-695.

**Introduction**	**Y/N**
1. Need for the curriculum	Y
2. Review of relevant literature, theories, models, or published curricula	Y
3. Unique contribution of the curriculum to the literature	Y
**Curriculum Development**	**Y/N**
4. Purpose/goals of the curriculum	Y
5. Outcome-based learning objectives	Y
6. Target population of learners	Y
**Curriculum implementation**	**Y/N**
7. Instructional setting for curriculum delivery	Y
8. Resources for implementing the curriculum	Y
9. Description of instructional methods	Y
10. Methods to evaluate achievement of outcome-based learning objectives	Y
11. Origin of evaluation instrument(s)	Y

Results	Y/N
12. Number of learners participating in the curriculum	Y
13. Number of participants included in the evaluation	Y
14. Evidence of achievement of outcome-based learning objectives	Y
**Discussion**	**Y/N**
15. Summary of findings	Y
16. Interpretation of findings in relation to the existing literature	N
17. Lessons learned from the implementation of the curriculum	N
18. Limitations of the evaluation of the curriculum	Y
19. Describes future implications of the curriculum	Y
